# Lipid–oligonucleotide conjugates for bioapplications

**DOI:** 10.1093/nsr/nwaa161

**Published:** 2020-07-09

**Authors:** Xiaowei Li, Kejun Feng, Long Li, Lu Yang, Xiaoshu Pan, Hoda Safari Yazd, Cheng Cui, Juan Li, Leonid Moroz, Yujia Sun, Bang Wang, Xiang Li, Tong Huang, Weihong Tan

**Affiliations:** Center for Research at Bio/Nano Interface, Department of Chemistry and Department of Physiology and Functional Genomics, Health Cancer Center, UF Genetics Institute and McKnight Brain Institute, University of Florida, Gainesville, FL 32611–7200, USA; Center for Research at Bio/Nano Interface, Department of Chemistry and Department of Physiology and Functional Genomics, Health Cancer Center, UF Genetics Institute and McKnight Brain Institute, University of Florida, Gainesville, FL 32611–7200, USA; School of Chemistry and Materials Engineering, Huizhou University, Huizhou 516007, China; Center for Research at Bio/Nano Interface, Department of Chemistry and Department of Physiology and Functional Genomics, Health Cancer Center, UF Genetics Institute and McKnight Brain Institute, University of Florida, Gainesville, FL 32611–7200, USA; Center for Research at Bio/Nano Interface, Department of Chemistry and Department of Physiology and Functional Genomics, Health Cancer Center, UF Genetics Institute and McKnight Brain Institute, University of Florida, Gainesville, FL 32611–7200, USA; Center for Research at Bio/Nano Interface, Department of Chemistry and Department of Physiology and Functional Genomics, Health Cancer Center, UF Genetics Institute and McKnight Brain Institute, University of Florida, Gainesville, FL 32611–7200, USA; Center for Research at Bio/Nano Interface, Department of Chemistry and Department of Physiology and Functional Genomics, Health Cancer Center, UF Genetics Institute and McKnight Brain Institute, University of Florida, Gainesville, FL 32611–7200, USA; Center for Research at Bio/Nano Interface, Department of Chemistry and Department of Physiology and Functional Genomics, Health Cancer Center, UF Genetics Institute and McKnight Brain Institute, University of Florida, Gainesville, FL 32611–7200, USA; Molecular Science and Biomedicine Laboratory (MBL), State Key Laboratory for Chemo/Bio- Sensing and Chemometrics, College of Chemistry and Chemical Engineering, College of Biology, and Aptamer Engineering Center of Hunan Province, Hunan University, Changsha 410082, China; Institute of Cancer and Basic Medicine (IBMC), Chinese Academy of Sciences; The Cancer Hospital of the University of Chinese Academy of Sciences, Hangzhou 310022, China; Molecular Science and Biomedicine Laboratory (MBL), State Key Laboratory for Chemo/Bio- Sensing and Chemometrics, College of Chemistry and Chemical Engineering, College of Biology, and Aptamer Engineering Center of Hunan Province, Hunan University, Changsha 410082, China; Center for Research at Bio/Nano Interface, Department of Chemistry and Department of Physiology and Functional Genomics, Health Cancer Center, UF Genetics Institute and McKnight Brain Institute, University of Florida, Gainesville, FL 32611–7200, USA; Center for Research at Bio/Nano Interface, Department of Chemistry and Department of Physiology and Functional Genomics, Health Cancer Center, UF Genetics Institute and McKnight Brain Institute, University of Florida, Gainesville, FL 32611–7200, USA; Molecular Science and Biomedicine Laboratory (MBL), State Key Laboratory for Chemo/Bio- Sensing and Chemometrics, College of Chemistry and Chemical Engineering, College of Biology, and Aptamer Engineering Center of Hunan Province, Hunan University, Changsha 410082, China; Center for Research at Bio/Nano Interface, Department of Chemistry and Department of Physiology and Functional Genomics, Health Cancer Center, UF Genetics Institute and McKnight Brain Institute, University of Florida, Gainesville, FL 32611–7200, USA; Center for Research at Bio/Nano Interface, Department of Chemistry and Department of Physiology and Functional Genomics, Health Cancer Center, UF Genetics Institute and McKnight Brain Institute, University of Florida, Gainesville, FL 32611–7200, USA; Center for Research at Bio/Nano Interface, Department of Chemistry and Department of Physiology and Functional Genomics, Health Cancer Center, UF Genetics Institute and McKnight Brain Institute, University of Florida, Gainesville, FL 32611–7200, USA; Center for Research at Bio/Nano Interface, Department of Chemistry and Department of Physiology and Functional Genomics, Health Cancer Center, UF Genetics Institute and McKnight Brain Institute, University of Florida, Gainesville, FL 32611–7200, USA; Molecular Science and Biomedicine Laboratory (MBL), State Key Laboratory for Chemo/Bio- Sensing and Chemometrics, College of Chemistry and Chemical Engineering, College of Biology, and Aptamer Engineering Center of Hunan Province, Hunan University, Changsha 410082, China; Institute of Cancer and Basic Medicine (IBMC), Chinese Academy of Sciences; The Cancer Hospital of the University of Chinese Academy of Sciences, Hangzhou 310022, China

**Keywords:** lipid–oligonucleotide, DNA nanostructure, biosensor, biomedicine, bioanalysis, nanoreactor

## Abstract

Lipid–oligonucleotide conjugates (LONs) are powerful molecular-engineering materials for various applications ranging from biosensors to biomedicine. Their unique amphiphilic structures enable the self-assembly and the conveyance of information with high fidelity. In particular, LONs present remarkable potential in measuring cellular mechanical forces and monitoring cell behaviors. LONs are also essential sensing tools for intracellular imaging and have been employed in developing cell-surface-anchored DNA nanostructures for biomimetic-engineering studies. When incorporating therapeutic oligonucleotides or small-molecule drugs, LONs hold promise for targeted therapy. Moreover, LONs mediate the controllable assembly and fusion of vesicles based on DNA-strand displacements, contributing to nanoreactor construction and macromolecule delivery. In this review, we will summarize the general synthesis strategies of LONs, provide some characterization analysis and emphasize recent advances in bioanalytical and biomedical applications. We will also consider the relevant challenges and suggest future directions for building better functional LONs in nanotechnology and materials-science applications.

## INTRODUCTION

Besides carrying genetic information, the programmability and self-recognition properties of DNA have shown great promise in promoting DNA nanotechnology for applications in biochemistry and materials sciences. The only limitation to building desired systems is the necessary reliance on pure DNA base pairing. Therefore, the combination of DNA and hydrophobic molecules has attracted attention as an important approach to increase the complexity of built DNA architectures. Here, hydrophobic groups could non-covalently protrude into the DNA backbone and form attractive bulk films, liquid crystals or hydrogels [1]. At the same time, chemically coupling oligonucleotides (ONs) with functional hydrophobic groups, such as polymers and lipids, have also been widely demonstrated [2].

Lipids are indispensable cell bilayer components in biological living systems and are essential for nutrient transport and cell-signaling processes. Lipid formulations are known to increase the solubilization of anticancer agents and have been utilized as carriers in dozens of drugs approved by the US Food and Drug Administration (FDA) [3]. For example, some peptides have been improved with better pharmacokinetics by using lipid carriers as protection against degradation in the gastrointestinal tract. By taking advantage of lipids, lipid-tagged ONs have also represented unique properties in the design of various molecular materials since the first publication in 1989 [4]. They could form multiple structures with different compositions and sizes by tuning experimental conditions and self-properties of the two moieties [5]. Relying on their molecular-recognition ability, information-transfer capability and self-assembly property, the developed lipid–oligonucleotide conjugates (LONs) have been used as membrane-anchored biosensors [6] and also synthetic lipid membrane channels [7]. Research examining their applications in therapeutic strategies [8] and controllable nanoreactors [9] has also been reported.

In this review, we will highlight recent advances in employing lipids for ON modifications to realize a variety of molecular-engineering nanostructures. We will first introduce the current major strategies for preparing LONs. This will be followed by a description of their basic structure and characterization of their properties. Finally, we will discuss future directions for applying LON probes in the bioanalytical and biomedical areas.

## DESIGN OF LIPID–OLIGONUCLEOTIDE PROBES

### Preparation: strategies for the synthesis of lipid–oligonucleotide conjugates

**Figure 1.**
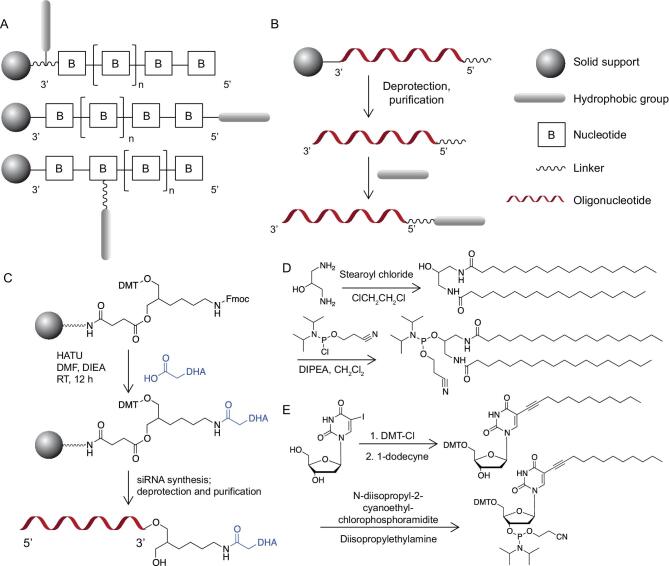
(A) Schematic illustration of the presynthetic-conjugation approach. Hydrophobic segments can be incorporated into the oligonucleotide at the 3^′^-termini or 5^′^-termini, or between consecutive nucleotides during solid-phase synthesis. (B) Schematic illustration of the postsynthetic-conjugation approach. (C)–(E) Revised schemes showing that the specific linker-modified oligonucleotides can be covalently coupled with the hydrophobic group after their respectively accomplished synthesis and purification. (C) DHA conjugation at the 3^′^-termini of ONs. (D) Synthesis of lipid phosphoramidite for 5^′^-termini conjugation of ONs. (E) Synthesis of DMT-on dodec-1-ynyl uridine for intrachain modification of ONs.

To achieve efficient synthesis of LONs, various approaches have been reported. Basically, two major strategies include the presynthetic approach and the postsynthetic approach (Fig. 1, Table 1). In the presynthetic-conjugation approach, the hydrophobic moiety-modified nucleotides are incorporated during the stepwise ON synthesis process. In the postsynthetic approach, the modification of lipid moiety is achieved after the complete synthesis and purification of the ON sequence. Since the postsynthetic conjugation is usually conducted in solution phase, allowing the separate lipid and ON strand to be assembled after respective syntheses, solid-phase-based presynthesis has a distinct advantage in terms of easier purification. However, the solubility and stability requirements and challenges encountered when introducing hydrophobic moieties directly into solid-phase ON synthesis have restricted its development. Herein, we discuss the recent efforts to construct LONs involving the two strategies. Advances in the preparation of LONs have also been highlighted in some recent reviews [5,10,11].

#### Presynthetic approach

So far, the conjugation of hydrophobic segments with ONs has mostly relied on solid-phase synthesis (SPS). Yet, the difficulties and cost of the required organic chemistries have posed challenges to hydrophobic modifications on ONs, essentially because such modifications need to withstand different conditions carried out during SPS. During the elongation of the ON sequence from the 3^′^-end to the 5^′^-end, substantial efforts have been made to incorporate hydrophobic modifications anywhere by using the three following methods (Fig. 1A): (i) hydrophobic parts are first functionalized on the 3^′^-terminal solid support; (ii) hydrophobic phosphoramidite building blocks are coupled onto the 5^′^-end of the ONs; and (iii) hydrophobic group-prefabricated nucleotides, or non-nucleoside building blocks, are inserted among consecutive nucleotides.

It is easier to introduce the hydrophobic group as a 5^′^-end phosphoramidite reagent as the ON chain extends from the 3^′^-end to the 5^′^-end, but many examples have been reported for the preparation of 3^′^-lipid-conjugated ONs [4,12]. The first report in the literature regarding 3^′^-cholesteryl-conjugated ONs was described by Letsinger and co-workers, in which the premade 3^′^-solid support-bound H-phosphonate derivative was tethered with a cholesteryl group by oxidative phosphoramidation [4]. Afterwards, more efforts were directed toward prefabricating solid supports and hydrophobic conjugates with specific linkers before their assembly [13,14]. One related work was reported recently by Nikan *et al.*, who conjugated docosahexaenoic acid (DHA) to the 3^′^-termini of siRNA sense strand via an amide bond, as shown in Fig. 1C [13]. This DHA conjugation enhanced the siRNA retention and silencing effect in mouse brain, showing the potential of modifying siRNA with hydrophobic groups for the treatment of neurodegenerative disease [13].

Besides 3^′^-conjugates bearing hydrophobic groups, it is noteworthy that many hydrophobic phosphoramidite building blocks are now available for coupling onto the 5^′^-end of ONs [15,16]. Since these phosphoramidites for 5^′^-conjugates typically do not possess a 4,4^′^-dimethoxytrityl (DMT) group, which is important for consecutive synthesis of ONs in the 3^′^–5^′^ direction, they could only be introduced at the 5^′^-terminus. For example, Nishina and co-workers prepared α-tocopherol phosphoramidite, combined it with siRNA at the 5^′^-termini and demonstrated efficient downregulation of the target mRNA *in vivo* [17]. Another straightforward procedure for creating 5^′^-LONs was reported by Godeau *et al.* Here, a dT-modified lipid phosphoramidite was synthesized by conjugating alkyne-labeled lipid with azido-dT via a ‘click-chemistry’ reaction and the resulting lipid phosphoramidite was further coupled to the 5^′^-end of the ON using SPS [18]. Our group has also been working on LONs for a long time. We have designed amphiphilic ON molecules by coupling constructed diacyllipid phosphoramidite onto the 5^′^-termini of ONs during SPS, as shown in Fig. 1D [16]. Importantly, the amphiphilic LONs-formed micelles are feasible for intracellular imaging and targeted gene therapy [19–21], which will be discussed further in the following sections.

Incorporation of the hydrophobic moieties into interchain positions of ONs is also an attractive method to construct LONs with the help of hydrophobic-group-premodified nucleoside or non-nucleoside DMT-on phosphoramidites. For instance, Guzaev *et al.* developed a non-nucleoside DMT-on phosphoramidite for multiple modifications of ON with octyl groups via potentially biodegradable ester moieties. These ester bonds could withstand the alkaline condition of DNA deprotection because of the proximate phosphate residues [22]. Additionally, Durand synthesized a dT-modified cholesterol phosphoramidite that could be positioned at any thymidine monomer site in the ON without interfering with base pairing [23]. The Herrmann group introduced another interesting prefabricated nucleoside phosphoramidite. As shown in Fig. 1E, the DMT-on dodec-1-ynyluracil nucleobase was synthesized and the resulting DNA amphiphiles were then assembled into micelles for virus capsid loading [24]. Other approaches were exploited as well, with many new hydrophobic group-functionalized phosphoramidites developed [25,26].

#### Postsynthetic approach

When using the postsynthetic-conjugation approach, it is required that certain reactive groups be attached to both ONs and lipids. The assembly of the ONs and lipid groups is then obtained after independent synthesis and purification. Some general methods have been developed to form different linkages connecting ONs and lipids in buffered solution, including thioether [27], phosphoester [28] and triazole [29] linkers. Raouane *et al.* developed an approach for the incorporation of the maleimide-modified natural lipid squalene with the thiolated siRNA sense strand via maleimide-sulfhydryl chemistry [27]. It was found that these thioether-linked amphiphilic molecules self-assembled into nanoparticles, showing strong inhibition towards tumor growth *in vivo* [27]. Recently, the Mirkin group demonstrated the synthesis of LONs using ‘click-chemistry’, in which the purified DBCO (Dibenzocyclooctyne)-terminated ON was reacted with azide-derivatized lipid chains [29]. Relying on the thermoresponsive ability of Pluronic F127, the authors cross-linked the micellar LONs for enhanced immunostimulation, while the non-cross-linked amphiphiles were removed by temperature transition [29]. A similar click-reaction-based strategy was reported by Awino and co-workers for the preparation of nucleic-acid nanocapsules with responsive drug-release ability [30]. They first let the alkyne-modified lipid moieties form a micellar structure. An esterified diazido cross-linker was then added to stabilize the micelle via ‘click-chemistry’. Finally, the thiolated ONs were coupled onto the remaining alkynes through a thiol-yne reaction [30].

### Characterization: analysis of LONs properties

Various designs have been introduced in the previous section to describe the efforts in conjugating hydrophobic groups with ONs. Yet, irrespective of the conjugation approach, each LON analogue has similar characteristics in a broad sense. Consisting of a specific ON head for target recognition (ONs, proteins or cells) and a hydrophobic tail for anchoring membranes or driving self-assembly, LONs have special properties owing to their two-part structures. Recognition ability and stability are the two main properties, as well as prerequisites, for LONs to function well in different applications and, to demonstrate this, several related studies will be discussed in this section.

#### Recognition property

**Figure 2.**
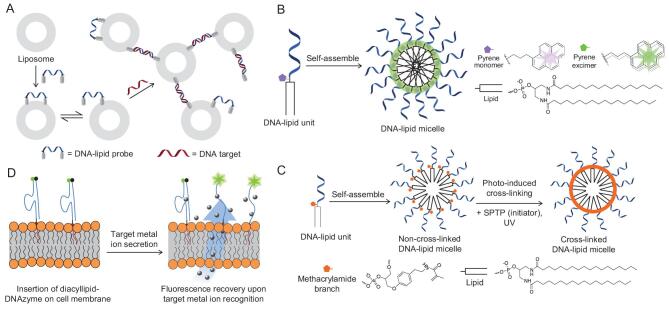
(A) Revised schematic illustration of liposome assembly controlled by the hybridization between DNA–lipid probes (blue) and targeted DNA (red). (B) Revised scheme illustrating the self-assembly of DNA–lipid micelles. The incorporated pyrene monomers aggregated and generated excimer-type fluorescence as a reporter in the micelle system. (C) Revised scheme illustrating the cross-linking of self-assembled DNA–lipid micelles after UV irradiation. (D) Revised schematic representation of DNAzyme sensor anchored on the cell membrane for real-time monitoring of metal ions in the cellular microenvironment.

ONs are unique information elements capable of specific recognition towards targets. Although fabricated with hydrophobic building blocks, the ON parts still retain their recognition property to aid in storing or transferring information with high fidelity in LONs. For example, Jacobsen *et al.* reported a lipophilic DNA-controlled liposome assembly for target-DNA detection by forming a specific DNA duplex via a Watson-Crick base pairing. As shown in Fig. 2A, by insertion of the four palmityl chain-labeled DNA strands in the same liposomes, the rigid dsDNA would favorably anchor on interliposomal membranes after hybridization with the external target-DNA sequence. The remarkable thermal transition and increase in particle size allowed accurate discrimination for targeted DNA, even with a single mismatch [31].

A similar idea was used for tethering lipid vesicles based on the recognition property of ON heads [32]. For instance, as reported by the Boxer group, the information encoded in the DNA–lipid conjugate in each preformed vesicle allowed the specific attachment to the complementary DNA sequence on the surface of a fluid-support lipid bilayer. This sequence-specific tethering model could be useful for the study of interactions between native membranes or cells with other components [32].

Most LONs could self-assemble in the water phase as a result of the hydrophobic effect caused by the lipid segments. Depending on the length of the ON part and the number of hydrophobic units, i.e. the balance between the electronic repulsions and hydrophobic effect, LONs could assemble to shift between spherical vesicles and micelles [33]. As presented by Thompson, LONs were demonstrated as programmable smart nanomaterials in response to the additional DNA strand. The recognition property of LONs enabled reversible hybridization with target-DNA strands, thereby driving the reversible vesicle–micelle phase switches [33].

Interestingly, the information-transfer ability of LONs not only facilitated the phase transitions in LONs-programmed vesicles, but could also help in the construction of lipid vesicles with designed geometry and controlled size. The Lin group recently published two papers reporting on the generation of liposomes by using DNA nanotemplating [34,35]. Basically, multiple single-stranded DNA handles that displayed on the DNA-origami ring or cage (DNA origami is a recent technique of folding DNA oligonucleotides to create various 3D nanoscale architectures for developing programmable DNA materials [36]) were hybridized with lipid-modified complementary DNA sequences. These lipid molecules then nucleated liposome formation with extra lipids added. As a result, liposomes with different complex membrane structures were generated based on defined DNA templates [34]. By applying this reconfigurable-membrane-engineering technique, a capable platform for studies of membrane mechanics, drug delivery across cell barriers and artificial organelles would be constituted in the future.

In addition to complementary sequences, nucleic-acid aptamers, known as chemical antibodies, are also widely used as target ligands because of their specific recognition property [37,38]. Aptamers are short, single-stranded DNA or RNA oligonucleotides generated by a process called SELEX (Systematic Evolution of Ligands by EXponential Enrichment) [38]. Over the past 30 years, many aptamers have been identified and could be approached via an aptamer database (e.g. Aptagen) and they have high binding affinity and specificity to many target molecules, including ions, small molecules, proteins and cells [39,40]. Aptamer–lipid conjugates have been exploited in our group over the years. One classical example to represent the recognition property of aptamer–lipid was from Wu *et al.*, who reported that amphiphiles could self-assemble into micelles and detect cancer cells with high specificity through the selective recognition of aptamers towards target cells [19]. More research about aptamer–lipid conjugates will be discussed in the following section.

#### Stability

In order to evaluate the stability of LONs, one of the easiest methods is to monitor the melting temperature (Tm) of the duplex formed between the target LON and its complementary sequence, as a higher Tm usually indicates the higher complementarity of the duplex [41]. One related study was reported by Letsinger *et al.*, who found that the appropriate positions of hydrophobic groups could facilitate base pairing between LONs. In particular, they found that a high Tm was exhibited when the base pairing happened in an unfavorable orientation, as both the poly-A and poly-T were modified with cholesteryl at their 5^′^-ends, indicating the strong hydrophobic forces required to stabilize the formation of a ‘wrong’ duplex [41].

Critical micelle concentration (CMC) is commonly used to characterize the assembly stability of LONs. When the concentration of LONs is above the CMC, LONs become thermodynamically willing to aggregate into the micelle structure to reduce free energy in the system. And it has been previously reported that a decrease in the CMC could help to enhance micelle stability, usually due to the longer hydrophobic lipid domain, which, however, would increase the micellar size and lower encapsulated drug performance *in vivo*, as the thermodynamic equilibrium of the micelle assembly would be hard to achieve [42]. Our group has utilized the fluorescence spectrum of pyrene molecules to determine the CMC of DNA micelle flares [16,43]. Basically, a pyrene phosphoramidite was incorporated into each LON amphiphile (Fig. 2B). Since multiple pyrene units in self-assembled ONs tend to aggregate and produce excimer-type fluorescence, the shift of fluorescence spectra from pyrene monomer to excimer versus the lowest LON concentration indicates the upper limit of the CMC. By using this method, it was determined that our micelle flares had a very low CMC, at ∼10 nM, suggesting the superior stability of the micelles independently of the effects of the ON length and temperature [43].

Apart from physical stability, the biostability of LONs is also significant, since many designs aim to apply the modified LONs to real biological environments. Accordingly, nuclease-resistance studies have been carried out to examine the stability of DNA–lipid micelles compared to single-stranded DNA using deoxyribonuclease I (DNase I) or serum proteins [43]. Free ON has been known to be quickly degraded in the presence of serum nucleases, with a half-life ranging from a few minutes to several hours. However, modifications on ONs such as peptide nucleic acids (PNAs) and locked nucleic acids (LNAs) have rendered significant nuclease resistance due to their unnatural entities-mediated low recognition from nuclease, with an increased half-life from hours to days [44,45]. In terms of LONs, fast micelle formation was also able to prevent degradation by nuclease, possibly resulting from the steric hindrance of the DNA corona. Subsequent studies in the Tan group further improved the physical and biological stability of DNA–lipid micelles [46–48]. Our most recent study reported the introduction of a methacrylamide unit between the DNA and lipid segments, which served to cross link the amphiphiles after self-assembly via photoinduced polymerization (Fig. 2C) [46]. Compared with traditional non-cross-linked micelles, the resulting micelles showed enhanced structural stability, as demonstrated by electrophoresis analysis, as well as excellent biostability after evaluation by DNase I and serum. This efficient covalent cross-linking method facilitated the construction of micelles and rigidified the structures without disturbing the specific recognition ability of the DNA aptamer [46].

With excellent recognition ability and stability, LONs and related micelle structures could be applied to various bioanalytical and biomedical applications. But, in order to achieve the desired goals, it is critical to consider the balance between increasing structural and probe stabilities (lowering CMC) and enhancing drug-loading or targeted-recognition abilities. As a consequence, better LONs should be designed with attempts at (i) stabilizing LONs by using covalent or non-covalent cross-linkers, or complexing with other fatty acids, or modifying ON heads with other architectures (e.g. cyclization [49]); (ii) improving targeting the hydrophilic head by either increasing quantities or exploiting more complex structures (e.g. molecular beacons giving ‘turn-on’ recognition signals); (iii) exploring more drug-loading possibilities, or combining drugs in the LON synthesis or cross-linking (e.g. nucleoside analogue clofarabine drug [50]), in terms of LON-based therapeutics.

## BIOANALYTICAL AND BIOMEDICAL APPLICATIONS OF DNA–LIPID PROBES

### Cell-membrane-anchored biosensors

The cell surface and cellular microenvironment comprise molecules expressed on the cell surface and various local biological species. This is the hub of cell–cell communication and cell signaling [51]. Extracellular signals from metal ions, pH, cytokines, growth factors and biomolecules themselves influence the activities of a key set of cell-surface receptors and downstream pathways, all constituting a complex network for sensing environmental changes, regulating cellular activities and maintaining homeostasis [52]. This microenvironment plays a pivotal role in regulating cellular activities, including metabolism, growth, development, differentiation and apoptosis [53]. Disturbance of the microenvironment may contribute, or relate, to disease progression, as manifested by inflammation, onset of tumorigenesis and metabolic diseases [54]. Insight into biomolecule distribution and interaction patterns in the local surroundings or on the live-cell membranes is critical to biological studies about cellular functions, disease diagnosis, as well as drug discovery. Powerful biosensing and bioimaging techniques would significantly improve our understanding and ability to manipulate biological mass dynamics and functions.

Recently, such technologies as mass spectrometry, Western blot and enzyme-linked immunosorbent assay (ELISA) have been developed for analysing purified molecules from cell-culture media and cell lysate [55]. However, most of these methods can only detect and measure bulk samples. While valuable, developing biosensing tools for molecules in native forms found in the live-cell microenvironment still encounters challenges. To address these engineering challenges, well-designed molecular sensors have been anchored onto the cell membrane, resulting in the performance of live-cell studies with high spatio-temporal resolution [56]. We recently developed varied LON structures in which the lipid moiety allows facile and efficient engineering of DNA-based sensors onto the cell phospholipid bilayer [6,56,57]. Compared to previous methods, lipid modification can be considered as a universal strategy without affecting cell physiology or sensor performance and no complicated processes are required.

#### Real-time monitoring of substances in the extracellular microenvironment

Some cellular substances, including protons, metal ions, small molecules and proteins, can affect cell functions and biological processes. Changes in the concentration of these substances are often associated with disease progression. Consequently, studying and monitoring these target molecules in real time would be beneficial to improve our ability to understand and manipulate biological systems. Currently, various technologies, such as flow cytometry and confocal laser-scanning microscopy, have been proposed to probe and image many kinds of events in the extracellular microenvironment. Qiu *et al*. [57] first reported a simple and universal strategy for real-time monitoring of extracellular metal ions based on a one-step insertion of a DNAzyme sensor on the cell-membrane surface after having modified a diacyllipid tail on DNA via SPS (Fig. 2D) [58]. Compared with other methods, this modification process does not require applying toxic chemical reactions [59], specific sites [60] or complicated manipulations [61]. Furthermore, the lipid-insertion strategy rarely affects the cell's physiological performance or sensing characteristics (stability and reliability). The experimental results also show that the self-assembled lipid-aptamer anchored on the cell surface has higher surface coverage and higher efficiency than can be achieved by covalent chemistry methods. The decoration strategy was shown to be desirable and reliable, offering a convenient tool for the detection of various targets by modifying different functional DNA probes on the cell membrane. However, irreversible cleavage of the substrate strand induced by the target does not readily accommodate a fluctuating monitoring signal.

**Figure 3.**
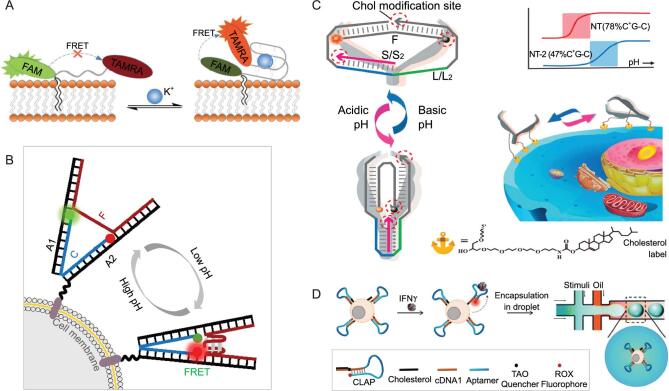
(A) Schematic illustration of the reversible membrane-immobilized K+ probe. Reprinted with permission from [63]. Copyright (2016) Royal Society of Chemistry. (B) Schemes of cell membrane-anchored DNA tweezers for extracellular pH monitoring. Reprinted with permission from [64]. Copyright (2018) American Chemical Society. (C) Reprinted with permission from [65]. Copyright (2018) American Chemical Society. (D) Schematic illustration of the T-cell-aptamer sensor for cytokine-secretion measurement. Reprinted with permission from [67]. Copyright (2017) Royal Society of Chemistry.

In fact, most analytical methods were designed to track target concentrations under ‘static’ conditions, while only a few have been designed to monitor the dynamics of the cell microenvironment [62]. Some reversible reactions, e.g. the formation of a quadruplex structure combined with potassium ions and a pH-sensitive i-motif sequence, may provide a strategy for dynamic monitoring in real time. Xiong *et al*. [63] designed a fluorescent probe based on a thrombin-binding aptamer (TBA). It could form a chair-type G-quadruplex structure in the presence of potassium ions and dynamically monitor them (Fig. 3A). The C18 diacyllipid linked with the TBA sequence from SPS was anchored to the cell membrane through hydrophobic interaction between the lipophilic tail and the cellular phospholipid layer. A FRET (Förster resonance energy transfer) pair offered signal information by the K^+^-induced structural change of the TBA. As the coordination between the TBA and K^+^ is fast and reversible, the fluorescence probe could be applied to real-time and reversible measurement of the fluctuation level of K^+^ in the complex cell microenvironment.

Similar designs were also applied to build some DNA-nanomachine-based biosensors, such as DNA tweezers, walkers and motors. Recently, DNA tweezers with a FRET pair as the signal reporter and cholesterol as the anchoring unit on the cell surface for extracellular pH monitoring were reported by both the Yao group (Fig. 3B) [64] and the Jiang group (Fig. 3C) [65]. In particular, the Jiang group investigated the number of cholesterol moieties anchoring on the cell surface. They found that multisite cholesterol anchoring could improve stability and prevent detachment of the DNA-sensing system from the cell-membrane surface. Moreover, they tuned the pH responsiveness by regulating the content of the C-G-C triplet and found that an enhanced response to higher acidity could be obtained with a higher percentage of the C-G-C triplet in the triplex motif. These cell-surface-anchored DNA tweezers have demonstrated a fast and reversible response upon extracellular pH change in real time. The strategies discussed here have potential for pH-related disease diagnostics and biomedical research.

In order to better understand the individual cellular, cell-to-cell variation and related processes, extensive interest has been generated in the field of single-cell research. Unlike ELISA, showing the advantage of analysing collective cellular properties [66], flow-cytometry assays could provide single-cell cytokine readouts. However, they could not be used to monitor cytokine production with high spatio-temporal resolution. In combination with a droplet-microfluidic system, Qiu *et al.* addressed this drawback by developing a simple, high-throughput and signal-on sensor to specifically monitor cytokine secretion (IFN γ) at the single-cell level, as shown in Fig. 3D [67]. The droplet-microfluidic system could generate picoliter water-in-oil droplets, which provided a versatile platform for the single-cell assay with low cost and high throughput. A cholesterol tail was attached to an aptamer probe and the two ends of the hairpin structure were separately labeled with a fluorophore and a quencher for recognition and sensing. They claimed that this strategy could be applied as a powerful and reliable molecular tool for studying immune response at the single-cell level.

#### Measuring biophysical events on cell surfaces

**Figure 4.**
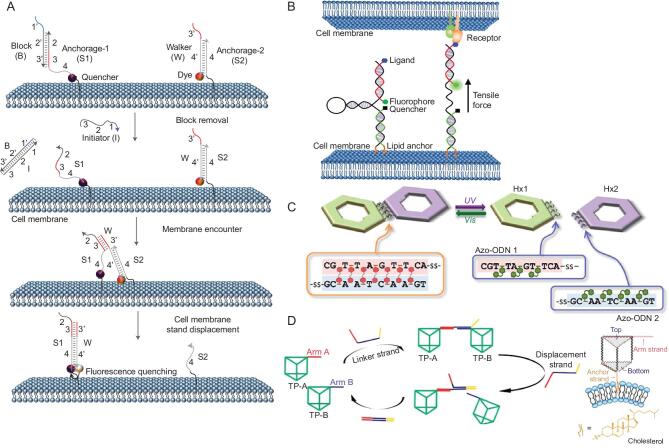
(A) Probing the dynamic and transient biomolecule-encounter events on a live cell surface with a DNA nanowalker. Reprinted with permission from [6]. Copyright (2017) Springer Nature. (B) Measuring tensile forces among cells with DNA-based membrane molecular probes. Reprinted with permission from [68]. Copyright (2017) American Chemical Society. (C) Assembly/disassembly actions of photoswitchable DNA-origami assembly. Reprinted with permission from [69]. Copyright (2014) American Chemical Society. (D) Reversible assembly processes of DNA triangular prisms on cell-mimicking giant membrane vesicles. Reprinted with permission from [70]. Copyright (2017) American Chemical Society.

The ability to quantitate the dynamic and transient molecular encounters of proteins and lipids is essential for understanding cell-membrane biophysics. However, transient encounter events between different cell-surface components are too fast (between the microsecond and millisecond ranges) to be detected by traditional analytical tools. Given the importance and challenge of such detection, a facile and efficient molecular probe compatible with standard fluorescence microscopy and flow cytometry is highly desired. This challenge has been overcome by a DNA walker reported by the Tan group (Fig. 4A) [6]. As biological programmable macromolecules, DNA-based walkers, which are able to walk directionally and progressively, have been recently developed to mimic biological protein motors in cargo transportation and biosynthesis. The DNA walker has also been found to be useful for probing dynamic and transient encounter events among biomolecules on the live-cell surface. To accomplish this, the DNA walker is anchored on the cell surface by lipid molecules on DNA strands; it then follows a track (cell membrane) while walking. DNA translocation on the DNA walker happens through a toehold-mediated strand displacement after two anchor sites (lipids) encounter by diffusion on the cell membrane. Because the accumulation signal is linearly correlated with the rate of lipid encounters, the walker has been successfully applied to measure the rapid encounter dynamics of two lipid molecules. By adopting a second-order-reaction model to calculate the encounter rates and 2D comparison of three kinds of membrane lipid-molecule anchors, the same lipid domains were found to have generally higher collision rates compared with heterogeneous encounters. The same strategy has also been extended to study cell-surface-receptor-encounter dynamics by using an aptamer-modified DNA nanowalker.

Mechanical forces regulate behavior such as cell migration, proliferation and differentiation, as well as guiding cell fate and many important cell-developmental processes. To date, however, force studies of intercellular and extracellular matrices and measurement at the cell–cell interface with traditional methods, such as monolayer stress microscopy and microfabricated cantilever array, have proven very challenging. Zhao *et al.* reported an efficient cell-membrane DNA probe to measure tensile forces among cells (Fig. 4B) [68]. Lipid-modified DNAs have been applied to efficiently anchor the tension probe onto the cell surface with high stability, while the other end of the probe was modified with cyclic Arg-Gly-Asp-d-Phe-Lys (cRGDfk) ligand, which readily linked to the second cell with the receptor. By using a distance-dependent FRET mechanism, the probe would have fluorescence output change when experiencing a change in forces between neighboring cells. The change of fluorescence resulted from the unfolding of the FRET pair on the DNA probe. With standard fluorescence microscopy, the probe's function has been successfully demonstrated and could be a potential approach to measuring various cellular processes.

#### DNA-nanoassembly behavior on cell-membrane mimicry

Thus far, DNA nanostructures, such as DNA walkers, tweezers and logic circuits, have been applied on the cell surface to perform intelligent tasks. In the meantime, massive studies have investigated the dynamic behavior of various DNA nanostructures on various types of cell-mimicking membranes, which may help to develop better understanding of membrane physicochemical properties and provide a significant impact on improving cell-surface-anchored DNA nanostructures. Suzuki *et al.* reported on photoswitchable DNA-origami assembly/disassembly using atomic-force microscopy (Fig. 4C) [69]. Their DNA origami was placed on a lipid bilayer surface with moderate mobility by cholesterol modification on the outer edges. One edge of the DNA origami was modified with sequence complementary azobenzene DNA strands and then used for dimer–monomer transition under UV- and visible-light irradiation. Assembly/disassembly behavior was successfully monitored by high-speed atomic-force microscopy in real time. Peng *et al.* investigated the assembly and disassembly processes of DNA triangular prisms on cell-mimicking giant membrane vesicles derived from mammalian cells (Fig. 4D) [70]. One arm of the 3D DNA prism was conjugated with a cholesterol anchor so that the construct could be efficiently localized on the giant vesicle surface. DNA-mediated strand hybridization and displacement were used to manipulate the dynamic behavior, as demonstrated by FRET. By regulating membrane-anchored DNA nanostructures, such methods can be applied for artificial-cell engineering and biomimetic nanotechnology.

### Targeted cancer theranostics

Owing to the similarity between conjugated lipid groups in LONs and the lipid bilayers in cell membranes, LONs have been recognized as having excellent biocompatibility and membrane permeability. After labeling specific fluorophores, LONs have shown compelling feasibility for intracellular imaging. Furthermore, effective delivery of encapsulated drugs or therapeutic ONs in the form of LONs has favored some administration concerns, including carrier safety and pharmacokinetic behavior. Thus, with versatile modifications, these hybrid LONs have been successfully extended to a larger perspective of applications in tumor imaging and targeted therapeutic strategies.

#### Imaging

Real-time imaging of living cells is essential for understanding life science—more specifically, the study of cancer progression. In this case, imaging tools with special properties would allow the visualization of both tumor sites in the body and the level of tumor behavior [71]. Yet, challenges remain owing to the lack of specific imaging probes against targeted cancer cells. As we discussed in the previous section, hydrophobic segments are good materials with which to penetrate cells with high affinity by having structures similar to those of cell membranes [72]. LONs could also incorporate nucleic-acid aptamers for specific target recognition. Therefore, efforts have been made to develop aptamer–lipid conjugate-based imaging probes for targeted cancer bioimaging.

**Figure 5.**
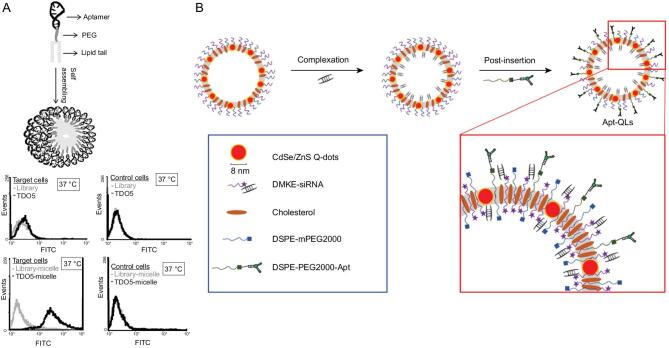
(A) Scheme for TDO5 aptamer–lipid micelle formation and flow-cytometry assay for binding profiles of aptamer only and aptamer micelles against target Ramos cells or control HL60 cells. Reprinted with permission from [19]. Copyright (2010) National Academy of Sciences. (B) Scheme for the synthesis of EGFR aptamer–lipid nanoparticles with quantum dots and loaded siRNAs. Reprinted with permission from [74]. Copyright (2017) Nature Publishing Group.

Aptamers in LONs can be functionalized with fluorophores to realize fluorescence-based optical imaging [21,46]. For example, our lab has labeled TDO5 aptamer with fluorescein to monitor the binding ability of aptamer–lipid micelles with targeted Ramos cells (a B-cell lymphoma cell line) (Fig. 5A) [19]. Interestingly, densely packed aptamers in the micelles resulted in a much better binding profile because of the multivalent effect; at the same time, the lipid part helped to achieve more rapid cell internalization. Thus, the substantially enhanced selective binding signal at a physiological temperature proved that the attachment of lipid groups to aptamers could generate unique nanomaterials for targeted cancer-cell imaging.

However, fluorescence imaging suffers from high background noise and superficial tissue penetration. To solve these problems, the utilization of quantum dots would be beneficial, as they were shown to have lower autofluorescence and deep inner-tissue penetration [73]. Kim *et al.* have built tumor-targeted lipid nanoparticles by combining EGF (epidermal growth factor) receptor (EGFR) aptamer–lipid conjugates and hydrophobic quantum dots responsible for target-cell recognition and fluorescence observation, respectively (Fig. 5B) [74]. The remarkable fluorescence signal from EGFR-positive cells, as well as *in vivo* tumor tissues, implied the potential of using this system for bioimaging in real surgery. By loading anticancer siRNAs, cancer-specific treatment was also expected [74]. Moreover, the Tang group fabricated aptamer-decorated self-assembled organic dots with aggregation-induced emission characteristics to enhance photostability and biocompatibility for better cancer-cell targeting and imaging [75].

#### Therapy

To realize targeted tumor therapeutics, studies involving the engineering of different drugs and drug-carrier systems have been carried out. The modes of delivering therapeutic agents must maintain efficacy while, at the same time, escaping potential off-target effects and immunogenicity [76]. LONs exhibit low cytotoxicity, controllable material size and available drug-loading sites; thus, by incorporating specific targeting ligands, different generations of LONs-based materials have been developed as efficient delivery vehicles for targeted cancer therapy.

##### Targeted drug delivery.

Small-molecule drugs have played the dominant role in new drug development in the pharmaceutical industries. And, in order to improve personalized treatment for patients, different combinations, formulations and delivery routes have been studied, as well as the incorporation of targeting ligands to provide drug delivery with specificity [77]. The ultimate goal of targeted drug delivery is to deliver a certain amount of therapeutic drugs with a prolonged circulation time, but smaller dosage frequency, to the targeted tumor area. One study direction has been focused on designing LONs-based nanoparticles as drug carriers, since hydrophobic side chains usually provide enough cavities to encapsulate small hydrophobic drug molecules.

**Figure 6.**
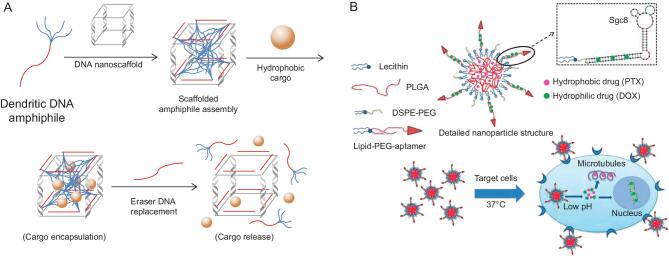
(A) Revised scheme showing spontaneous scaffolded dendritic chain–DNA amphiphile assembly upon adding specific DNA nanoscaffolds. The formed DNA cage could encapsulate small hydrophobic guest molecules and release them once destroyed by an eraser DNA sequence. (B) Schematic representation of self-assembled hybrid micelles for targeted co-delivery of two anticancer drugs. Reprinted with permission from [81]. Copyright (2014) Royal Society of Chemistry.

For example, the Sleiman group has built different LON assemblies by applying dendritic alkyl chain-based DNA amphiphiles to specific DNA nanoscaffolds [8,78]. As shown in Fig. 6A, guest molecules such as the hydrophobic dye Nile Red could be loaded in the inner space of the scaffolded assemblies and then be released upon specific DNA-sequence recognition and replacement. These LON amphiphiles-based 3D nanostructures were, therefore, capable of encapsulation and release of hydrophobic small-molecule drug cargos for future targeted-drug-delivery applications [8].

Studies employing real drug molecules were also reported. Here, either specific hydrophobic drugs targeting different diseases were loaded inside the hydrophobic core after LONs assembly or hydrophilic drugs bound with ONs [79–81]. Recently, Liu *et al.* delivered budesonide (sold as SYMBICORT, a glucocorticoid for treating chronic inflammation) through a UU11mer DNA–lipid micelle system, showing better drug solubility in the water phase and good anti-inflammatory activity [82]. In another study from Charbgoo *et al.*, doxorubicin (DOX), a widely used hydrophilic chemotherapy medication for treating multiple types of cancer, preferentially intercalated into double-stranded GC-rich regions in cholesteryl-modified MUC1 aptamer micelles, resulting in enhanced specific therapeutic effects with reduced DOX dosage in both *in vitro* and *in vivo* studies [80].

Our group has further demonstrated the co-delivery of two drugs into targeted cancer cells via aptamer–lipid micelles. In this construct, DOX was intercalated into modified sgc8 aptamer and the hydrophobic anticancer drug paclitaxel (PTX) was loaded into the lipid core (Fig. 6B) [81]. The hybrid nanoparticles with drug pairs were selectively transported into target CEM cells, rather than control Ramos cells, and demonstrated significant synergistic anticancer efficacy in the cytotoxicity assay [81]. Consequently, we could explore more advanced targeted-drug-delivery systems with other hydrophobic and hydrophilic anticancer drugs.

##### Gene therapy.

Gene therapy has offered promise for treating gene-related diseases. However, the introduction of specific genetic materials into targeted tumor sites is limited by several difficulties, such as cellular-membrane barriers, self-instability of materials and poor circulatory profiles [83]. To overcome these challenges, a variety of approaches have been reported, including the use of viral or non-viral vectors [84,85] and physical instruments, such as microinjection, electroporation and sonoporation [86]. Nevertheless, the toxicity, delivery efficacy and expense of these methods have been priority issues for further improvement before clinical applications.

Synthesis and modification simplicity, physiological safety and stability are some of the merits that have contributed to the success of LONs in the delivery of gene molecules for the induction or knock-down of specific gene expression. Our group has previously reported the synthesis of DNA–lipid micelle flares for intracellular mRNA detection and gene therapy [20]. The diacyllipid–molecular beacon conjugates self-assembled into micelle flares and showed efficient cellular uptake. Upon target-mRNA binding, the fluorophore on the molecular beacon was separated from the quencher molecule, resulting in the ‘turn-on’ fluorescence signal. The hybridization with target mRNA even induced a remarkable inhibition of cancer-cell proliferation, suggesting that simple modification with lipid afforded molecular beacons with enhanced cellular entry and targeted gene therapy [20].

Some researchers have directly attached hydrophobic domains to the antisense ONs in order to improve cell internalization and inhibition efficiency of target mRNA in the absence of additional transfection materials [15,87]. For instance, Rush and co-workers conjugated LNA with a hydrophobic tail to build spherical micellar nanoparticles, achieving rapid cell uptake and efficient target-mRNA regulation [15]. It is noteworthy that the densely displayed nucleic acids on the nanoparticle surface restricted degradation from enzymes and significantly increased the depletion ability towards targeted mRNA.

**Figure 7.**
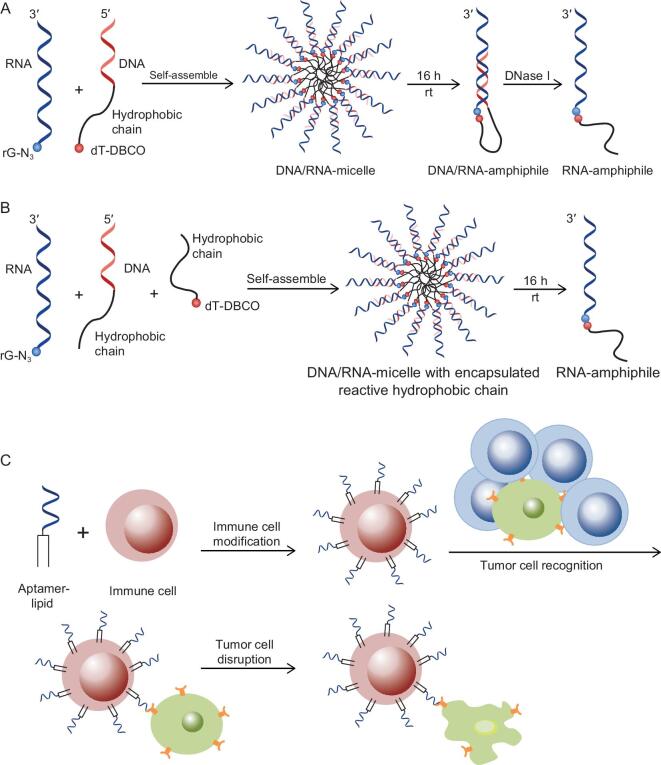
(A) and (B) Revised schemes of two methods for RNA-amphiphile synthesis. (A) RNA-N_3_ was hybridized with complementary DNA-amphiphile–DBCO, followed by the formation of DNA/RNA-amphiphile after self-assembly via ‘click-chemistry’. (B) Reactive DBCO-labeled hydrophobic chain was encapsulated in the DNA/RNA micelle, facilitating conjugation. (C) Revised scheme showing modification of immune cells with aptamers for specific recognition towards targeted cancer cells (green), rather than normal cells (blue), and subsequent cell–cell adhesion-induced cancer-cell disruption.

A more recent study from the Sleiman group reported two methods for conjugating specific RNA strands with hydrophobic chains, allowing the formation of RNA-amphiphiles and subsequent efficient gene-silencing capability [88]. As shown in Fig. 7A and B, by employing dibenzocyclooctyne group (DBCO)-labeled DNA-amphiphile as a template, azide-modified RNA was directly attached to the hydrophobic chain via ‘click-chemistry’ and the hybridized DNA could then be removed by adding DNase I. This interesting method provided a novel solution to the challenge of linking RNA strands with hydrophobic molecules, and the outcome has improved the efficacy of RNA therapeutics [88]. To further increase therapeutic efficiency, another study has also reported that the co-delivery of small-molecule anticancer drugs and nucleotide molecules simultaneously could show a synergistic effect for specific tumor treatment [89].

##### Immunotherapy.

Cancer immunotherapy, which is based on an antitumor immune response to regulate tumor behavior, has shown therapeutic efficacy in the fight against various malignancies [90]. Many approaches have been reported to boost the development of cancer immunotherapy, ranging from vaccine-based [91] or immunoregulatory cytokine-related designs [92] to engineering immune cells with specific ligands [29,93,94]. Among these strategies, immunoregulatory ONs represent strong immunostimulatory ability as a tumor suppressor, such as unmethylated cytosine-guanosine (CpG) DNA motifs and double-stranded RNA motifs [95]. Liu *et al.* in our group previously introduced the combination of synthetic CpG oligodeoxynucleotides (ODNs) with lipid domains where the lipid tail helped constructs to insert into cell membranes and promoted the association of CpG motifs with tumor cells over a prolonged time [94]. This strategy was investigated with both *in vitro* and *in vivo* studies and exhibited potential for localized immunotherapy.

In another interesting study, Xiong *et al.* engineered immunological effector cells with an aptamer for targeted cancer-cell recognition, bringing killer cells specifically to attack cancer cells, while, at the same time, minimizing side effects (Fig. 7C) [93]. The immune cells gained recognition ability after modification with aptamers through lipid-tail linkage. Then, cell–cell adhesion allowed an effective cytotoxic immune response to kill targeted cancer cells. This simple, but effective, method served as a redirected immunotherapy approach and was expected to employ more aptamers for a variety of cell-based immunotherapies in the future [93].

### LONs-mediated assembly and fusion

The hydrophobic tail of LON conjugates gives rise to hydrophobic–hydrophobic interactions among different types of membranes, such as cell membranes, vesicle membranes and hydrophobic-droplet-phase membranes. This sort of non-covalent interaction was depicted as an anchoring phenomenon and it garnered tremendous attention as it enabled facile decoration of nucleic acids on the cell, liposome and emulsion-droplet surface. By virtue of DNA reactions, including hybridization and displacement, functionalized vesicles, or droplets, are endowed with controllable assembly and fusion behaviors. In the past two decades, many studies have contributed to broadening the application of LONs to control vesicle or droplet interactions, including programmable assembly, controllable nanoreactors and membrane-fusion-induced delivery and biosensing [96–98].

#### Sequential assembly

**Figure 8.**
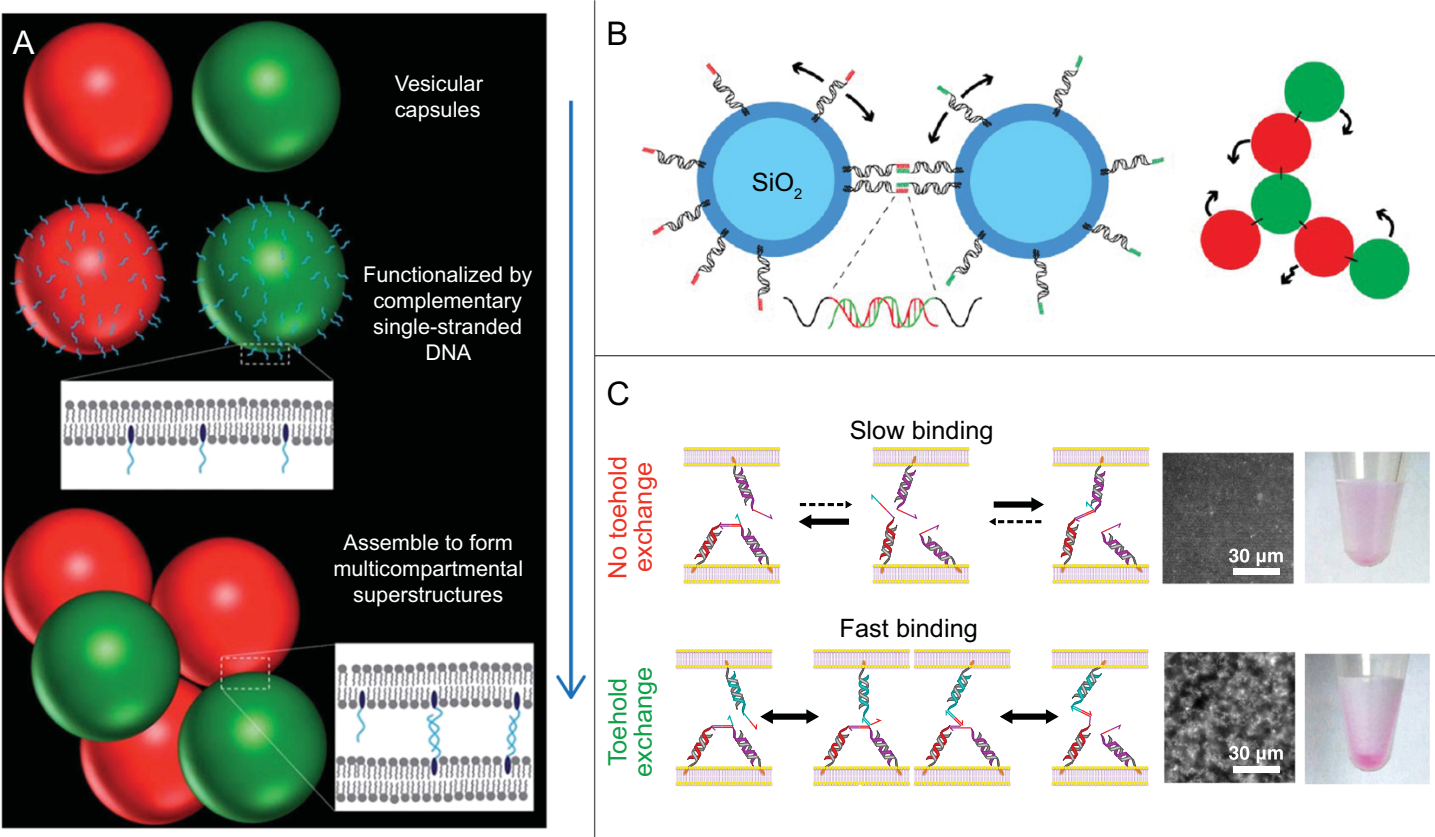
(A) Liposome assembly induced by specific hybridization of membrane-anchored DNA. Reprinted with permission from [100]. Copyright (2011) Royal Society of Chemistry. (B) Solid colloidal system composed of cholesterol-DNA-modified hard particles. Reprinted with permission from [101]. Copyright (2013) American Chemistry Society. (C) Kinetic liposome assembly mediated by DNA toehold exchange reaction. Reprinted with permission from [103]. Copyright (2016) American Chemistry Society.

Self-assembly behavior of nano/microscale-particle suspensions led to the design and creation of materials with tunable properties, especially through surface non-covalent interaction of particles in the bulky solution. DNA strands with specific hybridization and thermal reversibility have become a potent motif for particle-surface modification to enable assembly or aggregation. Soft materials, such as liposomes and oil-emulsion droplets, have a hydrophobic membrane, or surface, in aqueous buffer, which can then interact with the hydrophobic tail in LONs. In Vanderlick's work (Fig. 8A), lipid–DNA was first applied to the liposome surface in order to control vesicle association and population in solution [99,100]. Lately, this lipid–DNA-mediated interaction strategy has also been extended to hard nanomaterials, such as silicon [101]. A new solid colloidal system composed of hard and non-deformable particles was created by Mirjam *et al.* (Fig. 8B). The particles were modified with highly movable cholesterol–DNA conjugates on the surface. Thus, their colloids performed good association/dissociation transition under the control of DNA thermal reversible interaction.

In addition, the programmability of DNA has become a striking characteristic in the design and engineering of smart materials. Kinetic DNA hybridization, such as toehold reactions and strand-replacement reactions, allows sequential initiation, signaling and logical programming. The programmable potential of DNA by rational strand design is exemplified by nanodevices that employ DNA logic gates, DNA robotic motion and DNA computation circuits, as well as related concepts [102]. This attractive property was also transplanted into the design and engineering of smart materials, especially DNA-coated colloidal-system complexes. Assembly/disassembly behavior could also be programmed by lipid–DNA-mediated interaction by using DNA strands to connect the vesicles, or droplets, and by introducing sequential DNA-strand reactions. The complex phase behavior endorsed by DNA sequential displacement reactions in colloidal systems has enabled many innovative studies, such as ultrasensitive detectors and nanorobotic walkers. For example, Mognetti *et al.* designed a kinetic pathway to realize liposome assembly based on a toehold exchange mechanism among multiple types of surface-anchored lipid–DNA conjugates, as shown in Fig. 8C. The rearrangement of liposomes was enabled by cascade DNA interaction without the need to thermally denature DNAs [103].

#### Bioinspired fusion mediated by LONs

**Figure 9.**
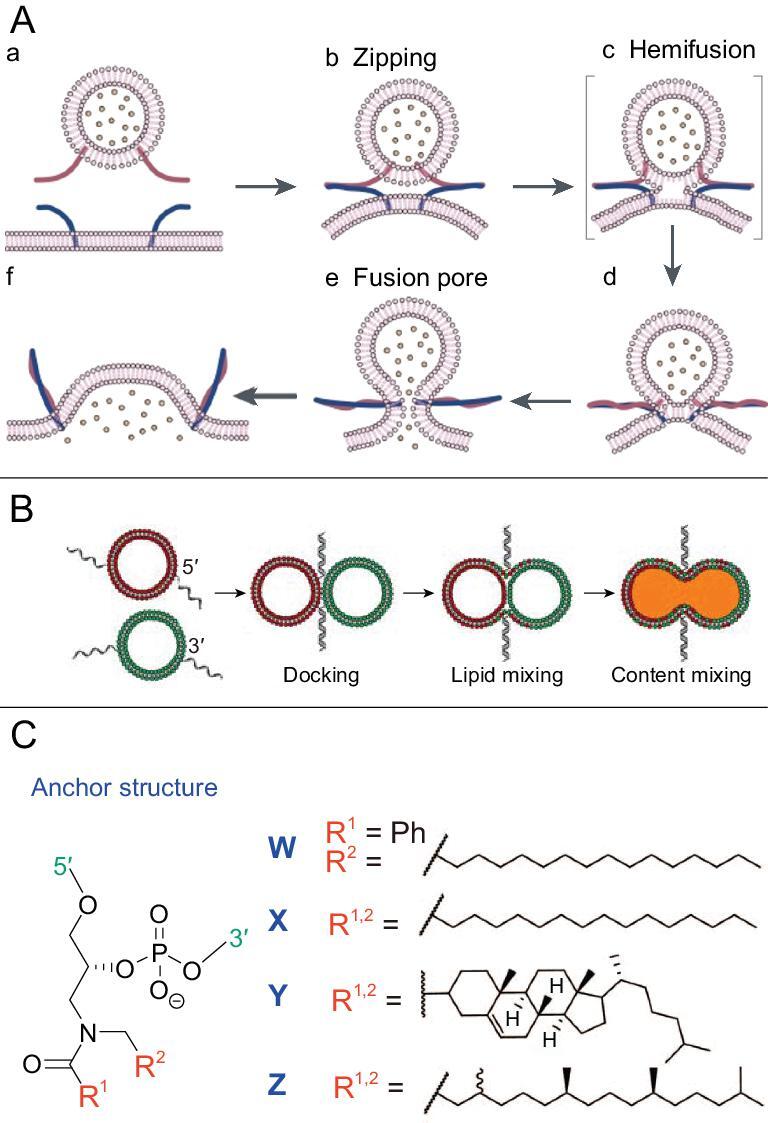
(A) Membrane-fusion process facilitated by SNARE protein. Reprinted with permission from [104]. Copyright (2001) Springer Nature. (B) Controllable vesicle fusion mediated by lipid DNA. Reprinted with permission from [106]. Copyright (2008) Springer-Verlag. (C) Different types of anchors in hydrophobically modified DNA. Reprinted with permission from [107]. Copyright (2017) Royal Society of Chemistry.

Although LONs can be applied to manipulate the complex assembly of compartmentalized vesicles, or droplets, to form colloidal architecture, another interesting model inspired by natural membrane fusion has attracted scientists’ attention to expand the function of lipid–DNA. Membrane fusion is a common phenomenon in cell communication, which plays an especially important role in neurotransmission, endocytosis and exocytosis. In the biological-membrane-fusion process, SNARE (Soluble N-ethylmaleimide-sensitive factor activating protein receptor) primarily mediates vesicle fusion whereby vesicles fuse with their target membrane-bound compartments, e.g. lysosome, according to the SNARE hypothesis [104]. It has been widely accepted that the SNARE complex was formed by a four-helix bundle between two membrane compartments (Fig. 9A), which could physically facilitate membrane interaction in the docking process [105]. Inspired by this zipper model, Boxer *et al.* developed a vesicle-fusion method mediated by lipid–DNA, as shown in Fig. 9B [106]. The fusion process operates via a mechanism similar to that of a LON-mediated assembly, which relies on hydrophobic tail-membrane anchoring and DNA hybridization, but it requires different membrane-anchoring geometry of strand pairs. Unlike the lipid–DNA-induced assembly in the previous section, whereby hydrophobic tails are always coupled on the 5^′^-end of DNA strands, the DNA hybrid in the fusion complex must have one strand coupled with the lipid at the 5^′^-end and the other at the 3^′^-end. Thus, hybridization can form into a zipper-like structure to pull the two vesicles into close apposition.

Boxer's controllable specific fusion method established the basis for using lipid–DNA to realize vesicle fusion and content mixing. Subsequent studies have dissected the physical process whereby factors, including membrane lipid components, temperature, DNA-strand length, linkers and hydrophobic anchors, were explored to improve this fusion model with higher fusion and content-mixing efficiency. Incorporation of a triethylene glycol spacer between the hydrophobic anchor and DNA was discovered to significantly increase fusion and content-mixing efficiency [107]. The most important factor was temperature, for which content-mixing efficiency was remarkable at 50°C with negligible leakage [107]. Cholesterol is another widely used alternative hydrophobic anchor owing to the commercial availability of cholesterol-coupling reagents for DNA modification. It was revealed that two cholesterol anchors are necessary to prevent DNA strands from shuttling to the same membrane, which could lead to membrane separation, rather than fusion. Other lipid anchors, such as phosphatidylcholine, phosphatidylethanolamine and sphingomyelin, were also explored, indicating the diversity of modification methods to enrich the lipid–DNA-mediated fusion model (Fig. 9C) [108,109].

**Figure 10.**
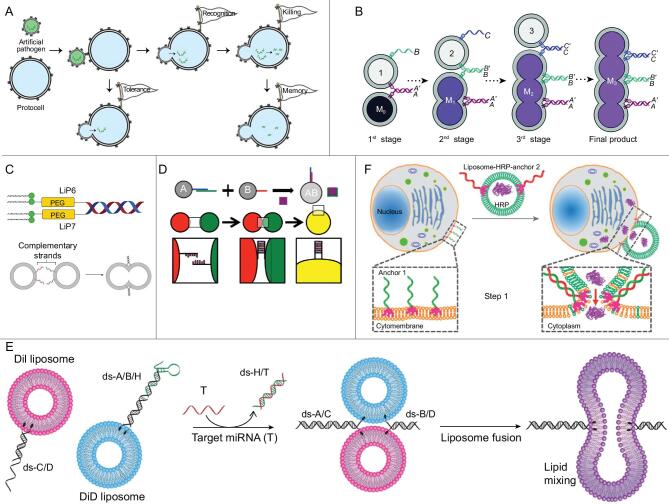
(A) Protocell communication mediated by cholesterol–DNA conjugate. Reprinted with permission from [110]. Copyright (2018) American Chemical Society. (B) DNA-controlled programmable liposome-fusion cascade. Reprinted with permission from [111]. Copyright (2017) John Wiley and Sons. (C) Lipidated peptide–DNA conjugate in the membrane-fusion model. Reprinted with permission from [112]. Copyright (2017) Royal Society of Chemistry. (D) Stimulus-responsive droplet fusion for biosensing in an emulsion system. Reprinted with permission from [113]. Copyright (2014) American Chemical Society. (E) MicroRNA detection by DNA-mediated liposome fusion. Reprinted with permission from [114]. Copyright (2018) John Wiley and Sons. (F) Protein delivery guided by DNA-programmed liposome–cell fusion. Reprinted with permission from [115]. Copyright (2018) Royal Society of Chemistry.

The liposome-based nanometer-sized bioreactor emerged as an artificial way to mimic biological compartmental synthesis in complex physical environment. However, the biggest limitation of the liposome nanoreactor is the impermeability of the bilayer membrane. For the standard liposome bulk solution, liposome vesicles must be homogeneously suspended with minimum association. On the contrary, to accelerate the reaction, fast aggregation and fusion among liposome units are desired at specific conditions. LON conjugates could perfectly resolve the conflict by enabling the specific recognizable liposome interaction so that on-demand reaction could occur when components from different liposome populations were mixed in the fusion process. Our group's recent work applied this strategy in building up the protocell communication, which successfully mimicked pathogen-cell immune response under cholesterol-DNA-mediated artificial protocell-membrane fusion (Fig. 10A) [110].

However, most bottom-up biological synthesis involves multistep reactions in which sequential mixing events from multiple compartments are required beyond the single-fusion event. To overcome this barrier, Vogel's team developed a DNA-programmed liposome-fusion cascade by introducing six different lipid–DNA conjugates [111]. As shown in Fig. 10B, four functionalized liposome populations were programmed to merge with three orthogonally hybridizing duplex sequences. The step-by-step fusion events allowed efficient content mixing and transfer of recognition units for the following stage. This artificial fusion cascade, as endorsed by DNA-coding versatility, is a promising platform for diverse applications, such as artificial protocell systems and liposome nanoreactor-based synthesis. In their next work, lapidated PNA were innovatively utilized to improve mismatch discrimination and stability toward enzymatic and chemical degradation of the liposome system (Fig. 10C) [112].

A similar strategy was also applied to the oil-in-water emulsion system to achieve rapid and controlled mixing of droplets in bulk solution. In Goodwin's work, a biosensor for thrombin was designed. Here, lipid–DNA-mediated fusion was triggered by competitive thrombin–aptamer binding [113]. Fluorescent-signal changes that resulted from component mixing, along with droplet fusion, could perform with high sensitivity down to 100 nM in only 15 minutes (Fig. 10D). Last year, another biosensing work using lipid–DNA liposome fusion successfully realized highly sensitive microRNA detection [114]. The flu biomarker miR-29a worked as a trigger to hybridize with the blocked hairpin, enabling the sticky ends of lipid–DNA to be released to hybridize with the complementary strands on another liposome population in a zipper-like manner. Based on the resulting increase in FRET signals from the liposome-fusion process, a low detection limit of 18 nM for the target miRNA was obtained (Fig. 10E).

With an increasing number of studies on DNA-mediated liposome fusion, the application of this model was further pushed into macromolecule delivery to increase cellular uptake and endosome release. The Fan group reported their efficient intracellular protein delivery using this lipid–DNA-programmed fusion strategy [115]. Encapsulated cargos could bypass the endocytic pathway and go directly into the cytoplasm via DNA-directed membrane fusion between liposomes and cells (Fig. 10F). This strategy presented potential in macromolecule delivery, gene editing and various translational applications.

## CONCLUSION AND FUTURE PERSPECTIVES

Since the first demonstration in the literature ∼30 years ago, LONs have become increasingly common in materials science and biotechnology (Table 2). Compared with other types of ONs, such as LNAs, PNAs and ON-polymers, LONs are more readily and efficiently synthesized and modified with lower cost using conventional chemistry; and they have better water solubility, drug-loading capability and penetration ability through the cell membrane, which are beneficial for theranostic applications without demanding extra drug vehicles or cellular-delivery strategies. With a diverse range of synthetic methods, lipids can be coupled onto ONs at different sites. The SPS approach enables automated and facile fabrication of LONs, while still requiring specific lipid phosphoramidites. In contrast, when combined with enzymatic reactions or click reactions, modified ONs and lipids could be linked in buffered solution, but may result in low yields with high cost. Therefore, efforts are still needed to overcome challenges in the preparation of LONs on a large scale with excellent reproducibility.

Solving the limitations involved in LONs stability and specificity remains another future task. Lipid digestion and ON degradation under specific enzymes or pH environments should be minimized to enhance the *in vivo* bioavailability and pharmacokinetics of transported therapeutic agents. Optimized LONs could be blended or engineered with other materials, such as biocompatible polymers and nanoparticles, to realize synergistic functionalities, which may further inhibit nanostructure disaggregation and drug precipitation. Besides increasing material types, combination with DNA-origami structures may facilitate the overall structural stiffness as well. Constructing covalent or stimuli-responsive linkers-based networks among LONs through different cross-linking methods may also provide a solution to stall the digestion of the formulation.

Although LONs have already been equipped with outstanding cellular permeability and drug-encapsulation capability, owing to the hydrophobicity and cell-membrane-mimicking property of lipids, continuous work to design more innovative LONs-based nanostructures with molecular-recognition and catalytic abilities is imperative to accelerate LONs applications in biosensing, biomedical diagnosis and treatment. The incorporation of functional ONs such as aptamer, siRNA and CpG could improve the targeting ability and help to increase the efficacy of therapeutic performance. Moreover, some other functional ON structures, including DNAzyme, G-quadruplex and i-motif, could be combined with lipids to develop new flexible composites to enable multiple and stimuli-responsive functionalities (e.g. precise drug release) by utilizing their conformational change and relationship with biomass in the microenvironment.

In addition to the theranostic applications of LONs, by adjusting the parameters of ONs and lipids, more future studies could emphasize on (i) investigating membrane biophysics, e.g. vesicle-fusion energy, which would facilitate the understanding of making biomimetic organelles and artificial cells; (ii) monitoring cellular behaviors, with improvement in cell-membrane surface-anchored DNA nanostructures to predict cellular processes and functions; (iii) exploring interactions between lipids and other molecules, such as proteins and sugars, to study their mechanisms, structures and functions in real cell membranes by generating biologically relevant membranes using specific LON structures; (iv) constructing nanoreactors, as LONs could form appealing containers for different chemical reactants to perform reactions more easily after the fusion process; (v) manipulating the assembly/disassembly of nanomaterials, as LONs have been used as potent templates to enable certain assembly through specific DNA-strand hybridization. Furthermore, studies investigating the length, coupling number and other molecular parameters of LONs should also be compared and optimized more deeply to construct a better nanostructure for different purposes.

It remains to be seen which LON formulation would be suitable for scale-up manufacture and for efficacy in different applications. More systematic studies from *in vivo* assessments in pharmacokinetics regarding the long-term toxicity of the physicochemical properties of LONs will further broaden LONs-based bioapplications. Improving LON constructs for clinical use should become a major future trend. Evaluations in animal models will also give guidance for new and better LON designs. It is believed that molecular engineering enabled by improved LON structures will lead to a promising expansion in bioanalytical and biomedical applications in the future.

**Table 1.** Summarized synthesis strategies of LONs.

Synthesis strategies	Characteristics	Hydrophobic-segment location	Conjugation methods	Selected references
Presynthetic approach	During ON synthesis; solid-phase synthesis	3^′^-end of ON	Specific linkers (e.g. amide bond)	[13,14]
		5^′^-end of ON	Phosphoramidite chemistry	[17]
		Between consecutive nucleotides	DMT-on phosphoramidite chemistry	[22–24]
Postsynthetic approach	After ON synthesis; solution-phase synthesis	5^′^-end of ON	Linkers (e.g. thioether, phosphoester, triazole)	[27–29]

**Table 2.** Summarized bioapplications of LON probes.

Bioanalytical and biomedical applications of DNA–lipid probes		DNA structure	Targets/goals	Cell types	Selected references
Cell-membrane-anchored biosensors	Real-time monitoring of substances in the extracellular microenvironment	DNAzyme	Mg^2+^	CEM	[57]
		Quadruplex	K^+^	CEM	[63]
		i-motif/Triplex	H^+^/OH^−^	CEM/HepG2	[64,65]
		Aptamer	Cytokine	T Cells	[67]
	Measuring biophysical events on cell surfaces	DNA walker	Transient encounter events measurement	Ramos/CEM	[6]
		DNA hairpin	Tensile-forces measurement	3T3	[68]
	DNA-nanoassembly behavior on cell-membrane mimicry	DNA origami	DNA assembly/disassembly investigation	Lipid bilayer surface	[69]
		Triangular prism		Cell-mimicking giant vesicles	[70]
Targeted cancer theranostics	Cancer imaging	Aptamer/micelle	Cancer-cell detection	Ramos	[19]
	Cancer therapy	DNA cage	Small-molecule drug delivery	−	[8]
		Micelle	Gene therapy	HeLa/HepG2	[88]
		Aptamer	Immunotherapy	NK/K562	[93]
LONs-mediated assembly and fusion	Sequential assembly	Complementary DNA	Liposome assembly	−	[100]
	Bioinspired fusion mediated by LONs	DNA-reaction network	Protocell communication	Artificial cells	[110]
		Complementary DNA	Nanoreactor	Giant unilamellar vesicles	[111,112]
